# Paddlewheel SBU based Zn MOFs: Syntheses, Structural Diversity, and CO_2_ Adsorption Properties

**DOI:** 10.3390/polym10121398

**Published:** 2018-12-17

**Authors:** Ting-Ru Lin, Cheng-Hua Lee, Yi-Chen Lan, Shruti Mendiratta, Long-Li Lai, Jing-Yun Wu, Kai-Ming Chi, Kuang-Lieh Lu

**Affiliations:** 1 Institute of Chemistry, Academia Sinica, Taipei 115, Taiwan; adjust331@gmail.com (T.-R.L.); sp123206505@gmail.com (C.-H.L.); yi.jen.lan@gmail.com (Y.-C.L.); shruti.mendiratta@gmail.com (S.M.); 2 Department of Chemistry and Biochemistry, National Chung Cheng University, Chiayi 621, Taiwan; 3 Department of Applied Chemistry, National Chi Nan University, Nantou 545, Taiwan; lilai@ncnu.edu.tw

**Keywords:** CO_2_ adsorption, metal–organic framework, secondary building unit, zinc

## Abstract

Four Zn metal–organic frameworks (MOFs), {[Zn_2_(2,6-ndc)_2_(2-Pn)]·DMF}*_n_* (**1**), {[Zn_2_(cca)_2_(2-Pn)]·DMF}*_n_* (**2**), {[Zn_2_(thdc)_2_(2-Pn)]·3DMF}*_n_* (**3**), and {[Zn_2_(1,4-ndc)_2_(2-Pn)]·1.5DMF}*_n_* (**4**), were synthesized from zinc nitrate and *N*,*N*′-bis(pyridin-2-yl)benzene-1,4-diamine (2-Pn) with naphthalene-2,6-dicarboxylic acid (2,6-H_2_ndc), 4-carboxycinnamic acid (H_2_cca), 2,5-thiophenedicarboxylic acid (H_2_thdc), and naphthalene-1,4-dicarboxylic acid (1,4-H_2_ndc), respectively. MOFs **1**–**4** were all constructed from similar dinuclear paddlewheel {Zn_2_(COO)_4_} clusters and resulted in the formation of three kinds of uninodal 6-connected non-interpenetrated frameworks. MOFs **1** and **2** suit a topologic 4^8^·6^7^-net with 17.6% and 16.8% extra-framework voids, respectively, **3** adopts a pillared-layer open framework of 4^8^·6^6^·8-topology with sufficient free voids of 39.9%, and **4** features a **pcu**-type pillared-layer framework of 4^12^·6^3^-topology with sufficient free voids of 30.9%. CO_2_ sorption studies exhibited typical reversible type I isotherms with CO_2_ uptakes of 55.1, 84.6, and 64.3 cm^3^ g^−1^ at 195 K and *P*/*P*_0_ =1 for the activated materials **1′**, **2′**, and **4′**, respectively. The coverage-dependent isosteric heat of CO_2_ adsorption (*Q_st_*) gave commonly decreased *Q_st_* traces with increasing CO_2_ uptake for all the three materials and showed an adsorption enthalpy of 32.5 kJ mol^−1^ for **1′**, 38.3 kJ mol^−1^ for **2′**, and 23.5 kJ mol^−1^ for **4′** at zero coverage.

## 1. Introduction 

Metal–organic frameworks (MOFs) are infinite arrays of metal ions connected by organic modules through coordination bonds [1]. Such materials possess fascinating structure variations and some can sustain considerable porosity and internal surface area, which is associated with intrinsic photo and magnetic properties. These properties have led to the evaluation of MOFs for various practical applications in gas adsorption/separation [2,3,4,5], ion exchange [6,7], sensing [8,9], catalysis [10], magnetism [11], drug delivery [12], etc. Thus, research on these kinds of superior materials have explosively advanced over the past decades and now have entered a new exciting stage. 

**Scheme 1:**
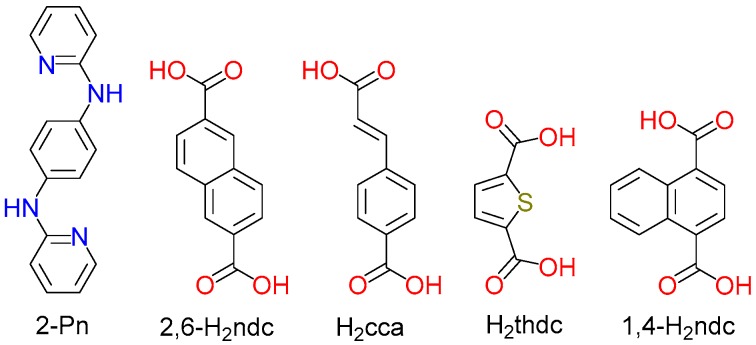
Molecular structures of organic ligands.

Crystalline MOF materials are usually constructed by metal clusters regarded as secondary building units (SBUs) and organic ligands [13]. Among them, paddlewheel type metal (II) carboxylates based on {M_2_(COO)_4_} SBUs have been advanced as a subclass of highly porous framework materials [14,15,16,17,18]. However, it is noted that a tiny change in chemical and/or physical factors might alter the connectivity and constitution of resultant metal clusters and also the orientation and ligating topology of organic links, and this would lead to varying framework topologies and functions of MOFs [19,20,21,22]. For example, Zn MOFs [Zn_2_(1,4-bdc)_2_(dabco)] and [Zn_2_(1,4-bdc)_2_(bpy)], where 1,4-H_2_bdc = benzene-1,4-dicarboxylic acid, dabco = diazabicyclo[2.2.2]octane, and bpy = 4,4′-bipyridine, both show **pcu** and **kag** net topologies [22]; such results are tentatively attributed to the degree of deviation of the conformations of the paddlewheel SBUs. Of note, this also suggests that judicially designed and modified MOFs may be promising porous materials for applications in gas adsorption such as carbon dioxide (CO_2_) capture [2,3,4,5,23,24,25,26,27,28,29,30]. For example, Zn MOF [Zn_2_(cca)(4-bpdb)_2_] (H_2_cca = 4-carboxycinnamaic acid, 4-bpdb = 1,4-bis(4-pyridyl)-2,3-diaza-1,3-butadiene) has shown noticeable CO_2_ uptakes of 54.2 cm^3^/g at 273 K and 38.9 cm^3^/g at 298 K [30]. As part of our ongoing efforts in the design and synthesis of functional crystalline materials [27,28,29,31], we herein report on the synthesis, structures and CO_2_ adsorption characteristics of porous Zn MOFs, {[Zn_2_(2,6-ndc)_2_(2-Pn)]·DMF}*_n_* (**1**, 2,6-H2ndc = naphthalene-2,6-dicarboxylic acid; 2-Pn *=* *N*,*N*′-bis(pyridin-2-yl)benzene-1,4-diamine, Scheme 1), {[Zn_2_(cca)_2_(2-Pn)]·DMF}*_n_* (**2**, H2cca = 4-carboxycinnamic acid), {[Zn_2_(thdc)_2_(2-Pn)]·3DMF}*_n_* (**3**, H2thdc = 2,5-thiophenedicarboxylic acid), and {[Zn_2_(1,4-ndc)_2_(2-Pn)]·1.5DMF}*_n_* (**4**, 1,4-H2ndc = naphthalene-1,4-dicarboxylic acid). All of these MOFs are constructed from similar paddlewheel {Zn_2_(COO)_4_} clusters but form different kinds of topologically 6-connected open structures that suit a 4^8^·6^7^ framework for **1** and **2**, a 4^8^·6^6^·8 framework for **3**, and a 4^12^·6^3^ (**pcu** type) framework for **4**. In addition, carbon dioxide (CO_2_)-capture properties of the activated materials were also studied. 

## 2. Experimental Section

### 2.1. Materials and Instruments 

The ligand 2-Pn was synthesized as reported previously [32,33]. Other chemicals were purchased commercially and used as received without further purification. Infrared spectra were recorded on a Perkin-Elmer Paragon 1000 FT-IR spectrophotometer (PerkinElmer, Taipei, Taiwan) in the region 4000–400 cm^−1^; abbreviations used for the IR bands are w = weak, m = medium, s = strong, and vs = very strong. Powder X-ray diffraction measurements were performed at room temperature using a Siemens D-5000 diffractometer (Siemens, Berlin, Germany) at 40 kV and 30 mA for Cu Kα radiation (*λ* = 1.5406 Å) with a step size of 0.02° in θ and a scan speed of 1 s per step size. Thermogravimetric analyses were performed under nitrogen with a Perkin-Elmer TGA-7 TG analyzer (PerkinElmer, Inc., Billerica, MA, USA). Elemental analyses were performed using a Perkin-Elmer 2400 elemental analyzer (PerkinElmer, Inc., Billerica, MA, USA). Brunauer–Emmett–Teller analyses and low-pressure carbon dioxide (CO_2_) adsorption measurements were investigated with a Micrometrics ASAP 2020 system using research grade carbon dioxide (99.9995% purity) as the adsorbate at 195 K, 273 K, and 298 K. Prior to analysis, the materials were immersed in EtOH for three days, then the EtOH-exchanged materials were loaded into a sample tube of known weight and activated at 120 °C under a high vacuum for about 24 h to completely remove guest solvent molecules. After activation, the sample and tube were re-weighed to determine the precise mass of the evacuated sample. 

### 2.2. Synthesis of {[Zn_2_(2,6-ndc)_2_(2-Pn)]·DMF}_n_ (**1**) 

A mixture of Zn(NO_3_)_2_·6H_2_O (29.7 mg, 0.10 mmol), naphthalene-2,6-dicarboxylic acid (2,6-H_2_ndc, 21.6 mg, 0.10 mmol), 2-Pn (12.7 mg, 0.050 mmol), EtOH (5 mL), and DMF (2 mL) were sealed in a closed glass tube and heated at 50 °C for 48 h. Deep purple block crystals of compound **1** were formed in 48% yield (21.5 mg, based on Zn^2+^). Solid products were isolated on a filter, and crystals were then washed with DMF and EtOH and dried in air. Anal. Calc. for C_43_H_33_N_5_O_9_Zn_2_: C 57.74; H 3.72; N 7.83. Found: C 57.88; H 3.82; N 7.86. IR (KBr pellet, cm^−1^): 3253(w), 3173(w), 3080(w), 2922(w), 1681(vs), 1594(vs), 1561(s), 1523(s), 1510(s), 1493(s), 1422(vs), 1364(s), 1331(s), 1297(s), 1263(m), 1212(s), 1119(m), 1099(m), 1063(w), 1015(vs), 945(w), 923(w), 891(m), 863(w), 832(s), 791(vs), 733(w), 713(s), 660(m), 644(m), 602(s), 537(s), 484(m), 464(w).

### 2.3. Synthesis of {[Zn_2_(cca)_2_(2-Pn)]·DMF}_n_ (**2**) 

A mixture of Zn(NO_3_)_2_·6H_2_O (29.7 mg, 0.10 mmol), 4-carboxycinnamic acid (H_2_cca,19.2 mg, 0.10 mmol), 2-Pn (12.7 mg, 0.050 mmol), EtOH (5 mL), and DMF (2 mL) were sealed in a closed glass tube and heated at 50 °C for 96 h. Deep purple block crystals of compound **2** were formed in 32% yield (13.5 mg, based on Zn^2+^). Solid crystalline product was isolated on a filter, washed with DMF and EtOH, and dried in air. Anal. Calc. for C_39_H_33_N_5_O_9_Zn_2_·H_4_O_2_: C 53.08; H 4.23; N 7.94. Found: C 53.08; H 3.85; N 8.03. IR (KBr pellet, cm^−1^): 3253(w), 3173(w), 3080(w), 2922(w), 1681(vs), 1594(vs), 1561(s), 1523(s), 1510(s), 1493(s), 1422(vs), 1364(s), 1331(s), 1297(s), 1263(m), 1212(s), 1119(m), 1099(m), 1063(w), 1015(vs), 945(w), 923(w), 891(m), 863(w), 832(s), 791(vs), 733(w), 713(s), 660(m), 644(m), 602(s), 537(s), 484(m), 464(w). 

### 2.4. Synthesis of {[Zn_2_(thdc)_2_(2-Pn)]·3DMF}_n_ (**3**) 

A mixture of Zn(NO_3_)_2_·6H_2_O (29.7 mg, 0.10 mmol), 2,5-thiophenedicarboxylic acid (H_2_thdc, 17.2 mg, 0.10 mmol), 2-Pn (13.3 mg, 0.050 mmol), MeOH (5 mL), and DMF (2 mL) were sealed in a closed glass tube and heated at 50 °C for 96 h. Deep purple block crystals of compound **3** were formed in 50% yield (23.7 mg, based on Zn^2+^). Solid crystalline products were isolated on a filter, washed with DMF and MeOH, and dried in air. Anal. Calc. for C_37_H_39_N_7_O_11_S_2_Zn_2_: C 46.65; H 4.13; N 10.29. Found: C 46.80; H 3.95; N 9.98. IR (KBr pellet, cm^−1^): 3303(w), 3103(w), 2930(w), 2844(w), 1683(s), 1638(s), 1615(s), 1580(s), 1529(m), 1514(m), 1472(s), 1448(s), 1379(vs), 1332(m), 1251(m), 1163(m), 1129(w), 1090(m), 1053(w), 1019(m), 893(w), 833(w), 806(m), 772(vs), 685(w), 660(m), 647(m), 555(w), 503(s). 

### 2.5. Synthesis of {[Zn_2_(1,4-ndc)_2_(2-Pn)]·1.5DMF}_n_ (**4**) 

A mixture of Zn(NO_3_)_2_·6H_2_O (59.4 mg, 0.20 mmol), naphthalene-1,4-dicarboxylic acid (1,4-H_2_ndc, 43.2 mg, 0.20 mmol), 2-Pn (25.9 mg, 0.10 mmol), and DMF (2 mL) were sealed in a closed glass tube and heated at 80 °C for 48 h. Deep purple block crystals of compound **4** were formed in 49% yield (45.4 mg, based on Zn^2+^). Solid crystalline products were isolated on a filter, washed with DMF and H_2_O, and dried in air. Anal. Calc. for C_44.5_H_36.5_N_5.5_O_9.5_Zn_2_: C 57.40; H 3.95; N 8.27. Found: C 57.88; H 3.82; N 7.86. IR (KBr pellet, cm^−1^): 3285(w), 2932(w), 1661(vs), 1602(vs), 1512(m), 1459(m), 1412(vs), 1365(vs), 1261(m), 1211(m), 1163(m), 1099(m), 1050(w), 1034(w), 1016(w), 868(w), 827(m), 798(s), 664(m), 582(m), 520(m). 

### 2.6. X-Ray Data Collection and Structure Refinement 

Suitable single crystals of **1**–**4** were mounted on the tip of a glass fiber and placed onto the goniometer head for indexing and intensity data collection using a Bruker Smart APEX 2 CCD diffractometer equipped with graphite-monochromatized Mo Kα radiation (λ = 0.71073 Å). Collection of intensity data was conducted at 150(2) K. Empirical absorptions were applied using the multiscan method [34]. The structures were solved by direct methods with the SHELXS-97 [35] program and refined against *F*^2^ by the full-matrix least-squares technique using the SHELXL-2014/7 [36] and WINGX [37] program packages. Nonhydrogen atoms were found from the difference Fourier maps, and most of them were treated anisotropically except where noted. Whenever possible, the amine hydrogen atoms were located on a difference Fourier map and fixed at the calculated positions, while the carbon-bound hydrogen atoms were geometrically placed and refined as a riding model. All of the hydrogen atoms were refined isotropically. In both **1** and **2**, the 2-Pn ligand was treated disorderedly over two positions with equal occupancy sites. For **2**, the cca^2−^ ligand was symmetrically disordered about an inversion center. For **4**, one lattice DMF molecule was disordered over two positions with occupancy sites of 0.55 and 0.45, respectively, whereas another was partially occupied with an occupancy site of 0.50. Some of the disordered atoms in **1** and **2** and lattice DMF molecules in **4** were refined isotropically. For **1**–**3**, there were lattice solvent molecules in the voids that were highly disordered and very difficult to model properly, in addition to these well-modeled DMF molecules. PLATON/SQUEEZE routine [38] indicated the presence of 217, 184, and 149 electrons per unit cell for **1**–**3**, respectively, which, associated with the elemental and thermogravimetric (TG) analyses, suggested an approximate quantity of lattice solvent of one DMF molecule per formula in both **1** and **2**, and three DMF molecules per formula in **3**. CCDC 1879110 (**1**), 1879111 (**2**), 1879112 (**3**), and 1879113 (**4**) contain the supplementary crystallographic data for this paper. These data can be obtained free of charge from the Cambridge Crystallographic Data Centre via www.ccdc.cam.ac.uk/data_request/cif. Experimental details for X-ray data collection and the refinements are summarized in Table 1.

## 3. Results and Discussion 

### 3.1. Crystal Structures of {[Zn_2_(2,6-ndc)_2_(2-Pn)]·DMF}_n_ (**1**) and {[Zn_2_(cca)_2_(2-Pn)]·DMF}_n_ (**2**) 

**Figure 1:**
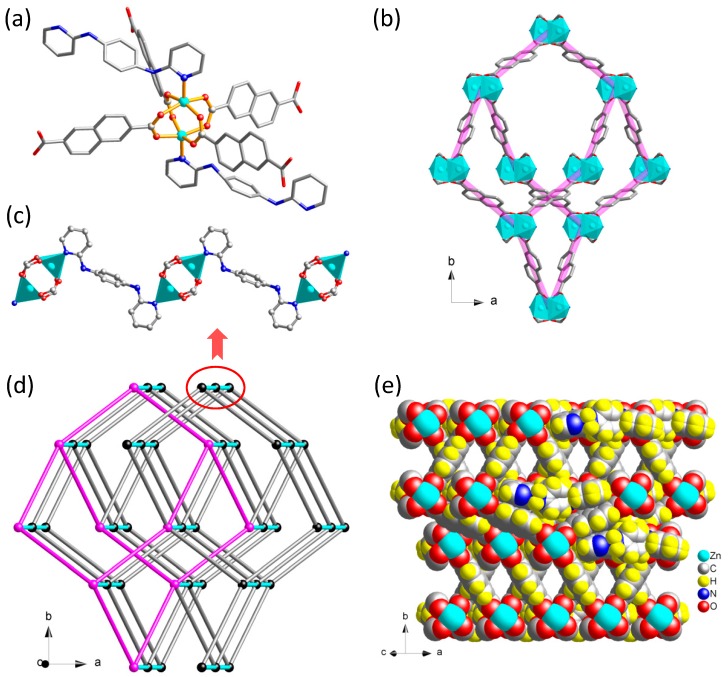
Molecular structure of **1**: (**a**) coordination environment of Zn(II) atom; (**b**) the Zn–2,6-ndc diamondoid network; (**c**) the linear chain consisting of {Zn_2_(COO)_4_} secondary building units (SBUs) and 2-Pn bridges; (**d**) the whole 6-connected non-interpenetrated framework with Schläfli symbol of 4^8^·6^7^ topology; (**e**) packing diagram viewed down the crystallographic [101] direction, showing the solvent-accessible channels.

Single-crystal X-ray diffraction analysis revealed that compound **1** crystallized in the monoclinic space group *C*2/*c*. The asymmetric unit consists of one Zn(II) center, two halves 2,6-ndc^2−^ ligands, and one half 2-Pn ligand. The Zn(II) center is coordinated with four basal oxygen atoms belonging to four distinct 2,6-ndc^2−^ ligands, and an apical nitrogen atom from a 2-Pn ligand, giving rise to a square pyramidal geometry (Figure 1a). In turn, the 2,6-ndc^2−^ ligand acts as a *μ*_4_-bridge linking four Zn(II) centers, in which both of the two carboxylate groups exhibit a *μ*_2_-*η*^1^:*η*^1^ coordination mode in a *syn*,*syn*-bridging bidentate manner. The 2-Pn ligand behaves as a *μ*_2_-bridge to link two Zn(II) centers through its two 2-pyridyl functions. The extended framework of **1** consists of dinuclear paddlewheel {Zn_2_(COO)_4_} clusters as SBUs, where the Zn···Zn separation is 3.0008(11) Å, and anionic 2,6-ndc^2−^ and neutral 2-Pn ligands as linear linkers. The anionic 2,6-ndc^2−^ ligands bridge {Zn_2_(COO)_4_} SBUs to form a three-dimensional (3D) Zn–2,6-ndc network suiting a 4-connected diamondoid (**dia**) topology with Schläfli symbol of 6^6^, in which the {Zn_2_(COO)_4_} paddlewheel SBU adopts a topologically distorted tetrahedral node instead of a common square planar node (Figure 1b). The neutral 2-Pn ligands bridge {Zn_2_(COO)_4_} SBUs to form a one-dimensional (1D) chain structure (Figure 1c), which intersects the Zn–2,6-ndc diamondoid network to give a whole 3D uninodal 6-connected non-interpenetrated framework with a Schläfli symbol of 4^8^·6^7^ topology (Figure 1d). When viewed down the crystallographic [101] direction, 1D channels were observed (Figure 1e), which suggests approximately 17.6% solvent-accessible volumes [39] to accommodate the lattice DMF molecules.

**Scheme 2:**
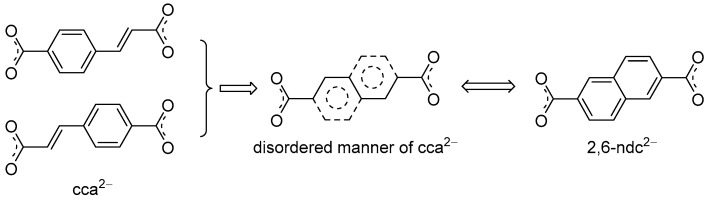
The similarity of spatial occupation between dislocated aromatic rings of cca^2−^ and 2,6-ndc^2−^.

Compound **2** crystallizes in the monoclinic space group *C*2/*c,* and the coordination environment of the Zn(II) center is shown in Figure S1. It is noted that the cca^2−^ ligand in **2** is symmetrically disordered about an inversion center, which results in similarity of spatial occupation between the dislocated aromatic rings of cca^2−^ and the well-located naphthalene ring of 2,6-ndc^2−^ (Scheme 2); this would lead to the formation of similar frameworks despite the use of different ligands. Thus, compound **2** has a molecular structure that is identical to that of **1** with 16.8% of the free extra-framework voids occupied by lattice DMF molecules. 

### 3.2. Crystal Structure of {[Zn_2_(thdc)_2_(2-Pn)]·3DMF}_n_ (**3**) 

**Figure 2:**
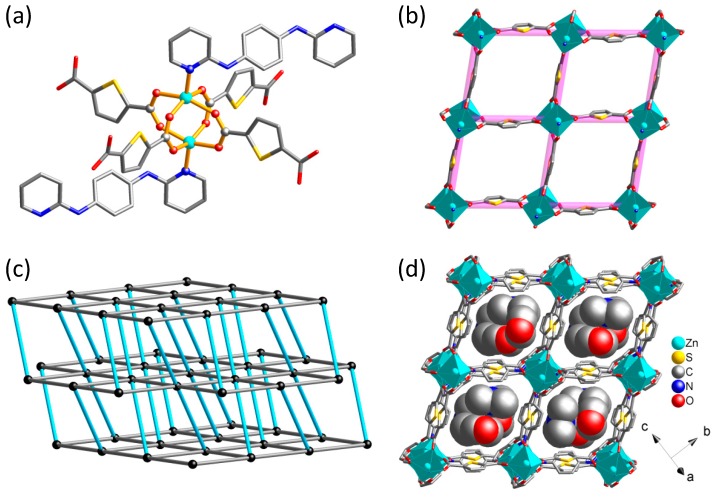
The molecular structure of **3**: (**a**) Coordination environment around Zn(II) center; (**b**) a single 2D Zn–thdc 4^4^-**sql** layer; (**c**) schematic representation of the whole 6-connected non-interpenetrated pillared-layer framework of 4^8^·6^6^·8-topology; (**d**) packing diagram viewed down the crystallographic [101] direction, showing lattice DMF molecules inside the solvent accessible pores.

Single-crystal X-ray diffraction analysis reveals that compound 3 crystallized in the monoclinic space group *C*2/*c*. The asymmetric unit consists of one Zn(II) center, one thdc^2−^ ligand, one half 2-Pn ligand, and one lattice DMF molecule. The Zn(II) center has a square pyramidal geometry, composed of four oxygen atoms of four distinct thdc^2−^ ligands in the basal plane and one nitrogen atom of a 2-Pn ligand at the axial position (Figure 2a). In turn, the thdc^2−^ ligand adopts a *μ*_4_-bridge mode to link four Zn(II) centers where each of the two carboxylate groups adopts a *syn*,*syn*-bridging bidentate to show a *μ*_2_-*η*^1^:*η*^1^ coordination mode. The 2-Pn ligand that serves as a *μ*_2_-bridge links two Zn(II) centers through its two 2-pyridyl functions. The extended framework of 3 is constructed by the dinuclear paddlewheel {Zn_2_(COO)_4_} SBUs, where the Zn···Zn separation is 3.0610(9) Å, and anionic thdc^2−^ and neutral 2-Pn linkers. Each {Zn_2_(COO)_4_} SBU is linked by four anionic thdc^2−^ ligands in a square planar manner to form a 2D gridlike Zn–thdc layer, which suits a topologic 4^4^-sql net (Figure 2b). The gridlike layers are connected by 2-Pn ligands as pillars in two different orientations, resulting in the formation of a new type of 3D pillared-layer framework. The whole 6-connected non-interpenetrated framework has a Schläfli symbol of 4^8^·6^6^·8 topology (Figure 2c). There are 39.9% solvent accessible volumes [39]. When viewed down the crystallographic [101] direction, the packing diagram shows 1D channels where lattice DMF molecules reside (Figure 2d). 

### 3.3. Crystal Structure of {[Zn_2_(1,4-ndc)_2_(2-Pn)]·1.5DMF}_n_ (**4**) 

**Figure 3:**
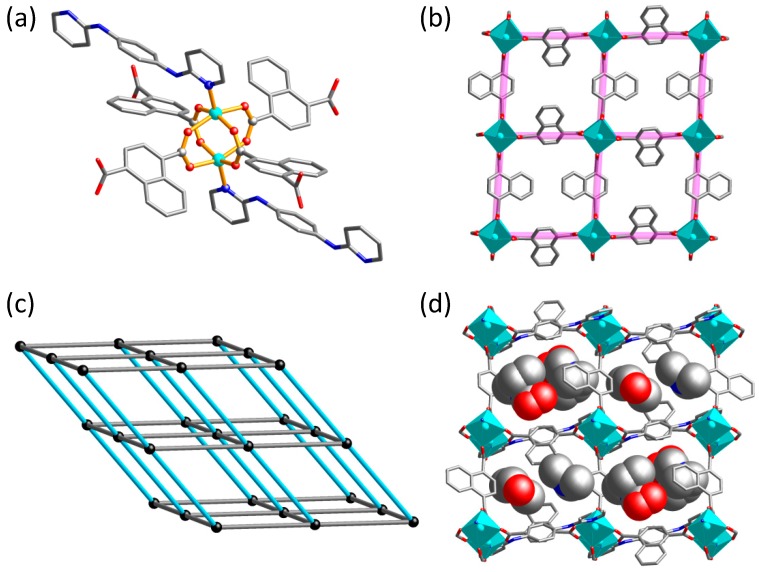
Molecular structure of **4**: (**a**) Coordination environment around Zn(II) center; (**b**) a single 2D Zn–1,4-ndc 4^4^-**sql** layer; (**c**) schematic representation of the whole 6-connected distorted **pcu**-type pillared-layer framework; (**d**) packing diagram viewed down the crystallographic *a*-axis, showing lattice DMF molecules inside the solvent accessible pores.

Single-crystal X-ray diffraction analysis revealed that compound **4** crystallizes in the triclinic space group *P*$\bar{1}$. The asymmetric unit consists of two Zn(II) centers, two 1,4-ndc^2−^ ligands, one 2-Pn ligand, and three partially-occupied lattice DMF molecules with occupancy sites of 0.55, 0.50, and 0.45, respectively. Both of the two distinct Zn(II) centers adopt a square pyramidal geometry, with four oxygen atoms belonging to four distinct 1,4-ndc^2−^ ligands occupying the basal plane and one nitrogen atom from a 2-Pn ligand occupying the apex (Figure 3a). The 1,4-ndc^2−^ ligand displays a bis(*syn*,*syn*-bridging bidentate) coordination mode (i.e., a *μ*_4_-bridge mode) linking four Zn(II) centers, where each of the two carboxylate groups show a *μ*_2_-*η*^1^:*η*^1^ coordination mode. The 2-Pn ligand serving as a *μ*_2_-bridge links two Zn(II) centers through its two 2-pyridyl functions. The extended framework of **4** consists of dinuclear paddlewheel {Zn_2_(COO)_4_} SBUs, where the Zn···Zn separations are 3.0157(13) and 3.0547(12) Å, and anionic 1,4-ndc^2−^ and neutral 2-Pn ligands as linear linkers. The {Zn_2_(COO)_4_} SBUs are a square planar node and are linked by the anionic 1,4-ndc^2−^ ligands to form a 2D Zn–1,4-ndc 4^4^-**sql** layer (Figure 3b), which extends to a 3D non-interpenetrated pillared-layer framework through the connection of 2-Pn pillars in the same orientation. The whole framework adopts a 6-connected distorted **pcu**-net with Schläfli symbol of 4^12^·6^3^ topology (Figure 3c). When viewed down the crystallographic *a*-axis, the packing diagram shows 1D channels having 30.9% solvent accessible volumes [39] that are occupied by lattice DMF molecules (Figure 3d).

### 3.4. Powder X-Ray Diffraction and Thermogravimetric Analysis 

**Figure 4:**
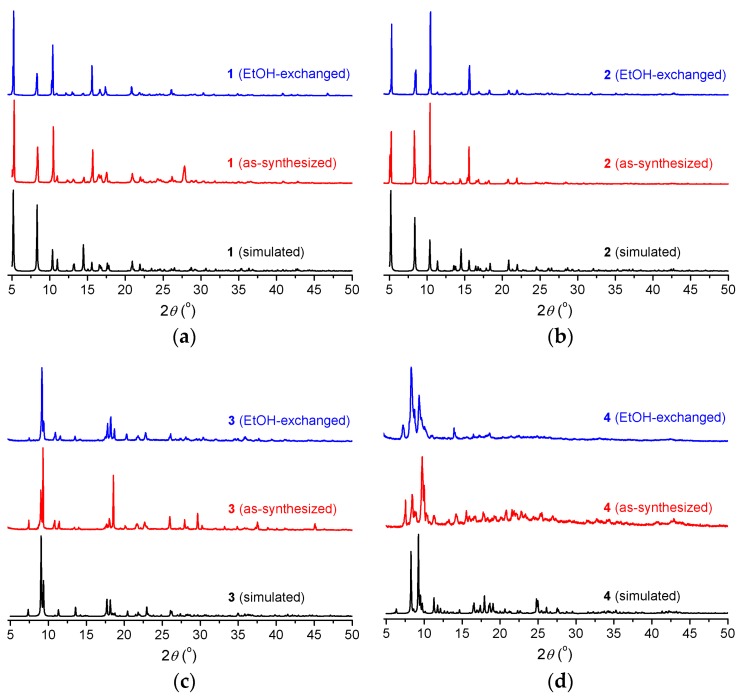
Powder X-ray diffraction (PXRD) patterns of simulated, as-synthesized, and EtOH-exchanged compounds (**a**) **1**, (**b**) **2**, (**c**) **3**, and (**d**) **4**.

Powder X-ray diffraction (PXRD) analysis was carried out to determine the purity of the compounds. The experimentally obtained patterns of compounds **1**–**3** matched well with the simulated patterns calculated from the single-crystal data (Figure 4), confirming phase purity. In addition, compounds **1** and **2** showed their stability in the atmosphere and in the presence of water over 4 days (Figure S2). Nevertheless, **4** showed an experimental pattern that did not match with the simulated pattern very well. A likely explanation for such differences is simply that the crystal packaging of the crystalline solid of **4**, after leaving the mother solution, might be distorted and/or partially collapse as a result of the release of the lattice DMF solvents before loading the sample for PXRD measurement and during the period of PXRD measurement. 

**Figure 5:**
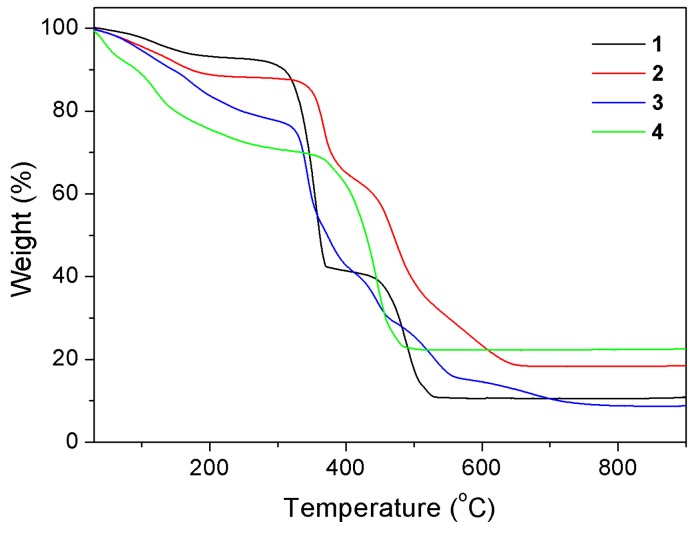
Thermogravimetric curves of compounds **1**–**4**.

Thermogravimetric (TG) analysis of **1** showed a weight loss between room temperature and ca. 175 °C, whereas that of **2** revealed a weight loss between room temperature and ca. 185 °C, corresponding to the escape of lattice DMF molecules (Figure 5). Both the solvent-free frameworks of **1** and **2** remained thermally stable up to a temperature of approximate 283 and 335 °C, respectively, followed by a decomposition process ending at approximately 530 and 650 °C, respectively. The TG curves of **3** and **4** both show that the lattice DMF molecules were gradually released upon heating from room temperature to about 318 and 360 °C, respectively, followed by a decomposition process ending at about 720 and 510 °C, respectively. 

### 3.5. CO_2_ Adsorption Properties 

**Figure 6:**
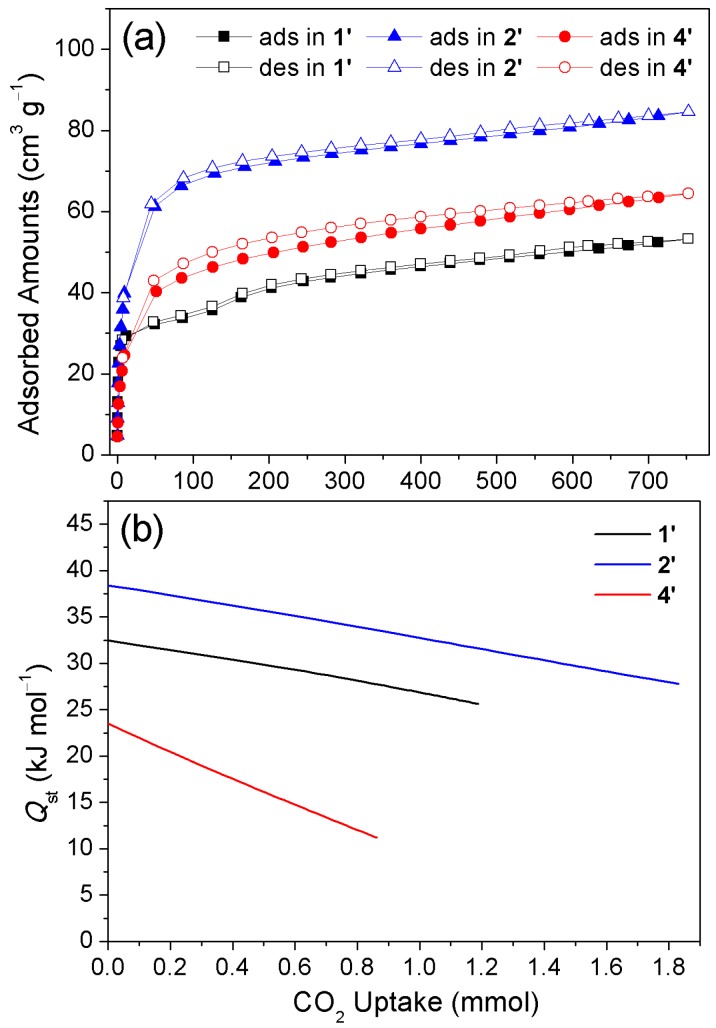
(**a**) CO_2_ adsorption isotherms of compounds **1′**, **2′**, and **4′** at 195 K. (**b**) Isosteric heat (*Q*_st_) of CO_2_ adsorption for **1′**, **2′**, and **4′**.

Prior to the gas adsorption experiments, ethanol was used to exchange guest DMF molecules. The TG curves of EtOH-exchanged materials show the success of complete substitution of DMF with EtOH according to the observations on both percentage and temperature of weight loss (Figure S3). PXRD patterns of EtOH-exchanged materials confirm the maintenance of structural integrity and crystallinity after solvent exchange for **1** and **2**, but show slight alternation of crystal packing after solvent exchange for **3** and **4** (Figure 4). The EtOH-exchanged materials of **1**, **2**, and **4** were degassed under a high vacuum at 120 °C for about 24 h to give the activated materials, denoted as **1′**, **2′**, and **4′**, for the use of CO_2_ capture studies. The structural integrity and crystallinity of these activated materials was maintained (Figure S2). Sorption studies showed that all the three activated materials exhibited as typical reversible type I isotherms with moderate CO_2_ uptakes of 55.1, 84.6, and 64.3 cm^3^ g^−1^ for **1′**, **2′**, and **4′**, respectively, at 195 K (Figure 6a). These values are comparable with those shown by other Zn MOFs (Table 2) [40,41,42,43,44,45]. For **1′** and **2′**, featuring identical framework topologies, the slight decrease of CO_2_ uptake for the former in comparison to that of the latter can be ascribed to its lower porosity. The Brunauer-Emmett-Teller (BET) analysis verified the lower surface area of **1′**, which was estimated to be 135 m^2^ g^−1^ (Langmuir surface area = 203 m^2^/g^−1^) compared to that of **2′** with a BET surface area of 233 m^2^ g^−1^ and Langmuir surface area of 347 m^2^ g^−1^. Compound **4′** showed a BET surface area of 173 m^2^ g^−1^ and Langmuir surface area of 230 m^2^ g^−1^, which is consistent with the CO_2_ uptakes among the three materials. To study the affinity between compounds and CO_2_ molecules, the coverage-dependent isosteric heat of CO_2_ adsorption (*Q_st_*) was calculated by the virial method based on the CO_2_ uptakes at 273 and 298 K (Figure S4), which gave commonly decreased *Q_st_* traces with increasing CO_2_ uptake for all the three materials (Figure 6b). At zero coverage, the adsorption enthalpy for **2′** exhibited a stronger binding affinity of 38.3 kJ mol^−1^ for CO_2_, compared to **1′** at 32.5 kJ mol^−1^ and **4′** at 23.5 kJ mol^−1^. However, the values are higher than that of liquefaction of CO_2_ (17 kJ mol^−1^) [46], suggesting significant interactions (possible dipole–quadruple interactions) between the framework surface and the CO_2_ molecules with the gas–gas affinity at higher temperature [27,28,29]. 

## 4. Conclusions 

In this work, we successfully synthesized four Zn MOFs featuring a topologic 4^8^·6^7^ net for **1** and **2**, a pillared-layer open structure of topologic 4^8^·6^6^·8 net for **3**, and a **pcu**-type pillared-layer open structure of 4^12^·6^3^-topology for **4**. These fascinating framework topologies were all constructed from similar 6-connected paddlewheel {Zn_2_(COO)_4_} SBUs connected by dicarboxylate links and 2-Pn bridges. As a result, these paddlewheel SBU-based Zn MOFs demonstrate cases showing interesting structure diversity that might be ascribed to the influences of dicarboxylate links with different spacers and 2-Pn bridges with varying ligating orientations. In addition, these MOFs also exhibited CO_2_ uptake abilities with noticeable binding affinity. 

## Figures and Tables

**Table 1:** Crystallographic data and structure refinement details for compounds **1**–**4** (excluding the squeezed solvent molecules).

Compounds	1	2	3	4
Empirical Formula	C_40_H_26_N_4_O_8_Zn_2_	C_36_H_26_N_4_O_8_Zn_2_	C_34_H_32_N_6_O_10_S_2_Zn_2_	C_44.5_H_36.5_N_5.5_O_9.5_Zn_2_
*M*_w_	821.39	773.35	879.51	931.03
Crystal system	Monoclinic	Monoclinic	Monoclinic	Triclinic
Space group	*C*2/*c*	*C*2/*c*	*C*2/*c*	*P* $\bar{1}$
*a*/Å	8.2847(17)	8.0307(16)	19.710(4)	10.697(2)
*b*/Å	34.127(7)	34.118(7)	15.618(3)	14.488(3)
*c*/Å	13.592(3)	13.527(3)	13.591(3)	14.912(3)
*α*/°	90	90	90	81.38(3)
*β*/°	93.66(3)	96.50(3)	106.47(3)	79.39(3)
*γ*/°	90	90	90	76.04(3)
*V*/Å^3^	3835.2(13)	3682.5(14)	4011.9(15)	2191.0(8)
*Z*	4	4	4	2
*T*/K	150(2)	150(2)	150(2)	150(2)
*λ*/Å	0.71073	0.71073	0.71073	0.71073
*D_cal_*_c_/g cm^−3^	1.423	1.395	1.456	1.411
*F*_000_	1672	1576	1800	956
*μ*/mm^−1^	1.307	1.357	1.360	1.157
*R*_1_^a^, *wR*_2_^b^ (*I* > 2*σ* (*I*))	0.0377, 0.1032	0.0426, 0.1208	0.0313, 0.0862	0.0574, 0.1674
*R*_1_^a^, *wR*_2_^b^ (all data)	0.0563, 0.1229	0.0480, 0.1253	0.0413, 0.1009	0.0915, 0.1909

^a^: *R*_1_ = $\sum \left|\left|F_{o}\right|–\left|F_{c}\right|\right|/\sum \left|F_{o}\right|$. ^b^: *wR*_2_ = $\{\sum \left[w{\left({F_{o}}^{2}–{F_{c}}^{2}\right)}^{2}\right]/\sum \left[w{\left({F_{o}}^{2}\right)}^{2}\right]{\}}^{1/2}$.

**Table 2:** Low-pressure CO_2_ adsorption analyses for a series of Zn metal–organic frameworks (MOFs) at 195 K.

Zn MOFs	CO_2_ Adsorption Amount (cm^3^/g)	Reference
[Zn_2_(2,6-ndc)_2_(2-Pn)] (**1′**)	55.1	this work
[Zn_2_(cca)_2_(2-Pn)] (**2′**)	84.6	this work
[Zn_2_(1,4-ndc)_2_(2-Pn)] (**4′**)	64.3	this work
[Zn_2_(L)(bpy)_2_](NO_3_)	50.6	[40]
[Zn_2_(L)(bpe)_2_](NO_3_)	74.9	[40]
[Zn_2_(L)(bpb)_2_](NO_3_)	146.9	[40]
[Zn_2_(btdc)(bpy)_2_] (3-fold interpenetration)	55	[41]
[Zn_2_(btdc)(bpy)_2_] (2-fold interpenetration)	201	[41]
[Zn_2_(bme-1,4-bdc)(dabco)_2_]	80.2	[42]
[Zn_2_(bpydb)(bpy)]	83	[43]
[Zn_2_(fma)(trz)_2_]	188.5	[44]
[Zn_2_(1,4-bdc)(trz)_2_]	119.4	[45]
[Zn_2_(NH_2_-1,4-bdc)(trz)_2_]	136.3	[44,45]
[Zn_2_(Br-1,4-bdc)(trz)_2_]	108.9	[44]
[Zn_2_(1,4-ndc)(trz)_2_]	86.88	[44]
[Zn_2_(oba)(trz)_2_]	70.68	[44]

Abbreviations: 2-Pn = *N*,*N*′-bis(pyridin-2-yl)benzene-1,4-diamine; bpb = 1,4-bis(4-pyridyl)benzene; bpe = 1,2-di(4-pyridyl)ethylene; bpy = 4,4′-bipyridine; dabco = diazabicyclo[2.2.2]octane; 1,4-H_2_bdc = benzene-1,4-dicarboxylic acid; NH_2_-1,4-H_2_bdc = 2-aminobenzene-1,4-dicarboxylic acid; Br-1,4- H_2_bdc = 2-bromobenzene-1,4-dicarboxylic acid; bme-1,4-H_2_bdc = 2,5-bis(2-methoxyethoxy)benzene-1,4-dicarboxylic acid; H_2_bpydb = 4,4′-(4,4′-bipyridine-2,6-diyl) dibenzoic acid; H_2_btdc = 2,2′-bithiophene-5,5′-dicarboxylic acid; H_2_cca = 4-carboxycinnamic acid; H_2_fma = fumaric acid; 1,4-H_2_ndc = naphthalene-1,4-dicarboxylic acid; 2,6-H_2_ndc = naphthalene-2,6-dicarboxylic acid; H_2_oba = 4,4′-oxybis(benzoic acid); H_4_L^+^ = 1,3-bis(3,5-dicarboxyphenyl)imidazolium; Htrz = 1,2,4-triazole.

## Supplementary Materials

The following are available online at http://www.mdpi.com/2073-4360/10/12/1398/s1, Figure S1: Coordination environment around Zn(II) center in compound **2**. The Zn···Zn separation in the {Zn_2_(COO)_4_} SBU is 2.9694(12) Å; Figure S2: PXRD patterns of compounds **1** and **2** as-synthesized in atmosphere for 4 days, in water for 4 days, and activated; Figure S3: Thermogravimetric curves of as-synthesized and EtOH-exchanged compounds **1**–**4**; Figure S4: CO_2_ adsorption isotherms and virial method fitting for *Q_st_* calculation.
