# Supplementary material for: Paddlewheel SBU based Zn MOFs: Syntheses, Structural Diversity, and CO2 Adsorption Properties
Source: Polymers (Basel). 2018 Dec 17;10(12):1398. doi: 10.3390/polym10121398 (PMC6401755; doi:10.3390/polym10121398)
Supplement: Supplementary file 1 [file polymers-10-01398-s001.pdf]

Electronic Supplementary Information for

Paddlewheel SBU based Zn MOFs: Syntheses, Structural Diversity, and CO_2 Adsorption Properties

Ting-Ru Lin,[1,2] Cheng-Hua Lee,[1] Yi-Chen Lan,[1] Shruti Mendiratta,[1] Long-Li Lai,[3] Jing-Yun Wu,[3*] Kai-Ming Chi,[2*] and Kuang-Lieh Lu[1*]

[1]Institute of Chemistry, Academia Sinica, Taipei 115, Taiwan

[2]Department of Chemistry and Biochemistry, National Chung Cheng University, Chiayi 621, Taiwan

[3]Department of Applied Chemistry, National Chi Nan University, Nantou 545, Taiwan

Corresponding Author: jyunwu@ncnu.edu.tw (J.-Y.W.), chekmc@ccu.edu.tw (K.-M.C.). kllu@gate.sinica.edu.tw (K.-L.L)

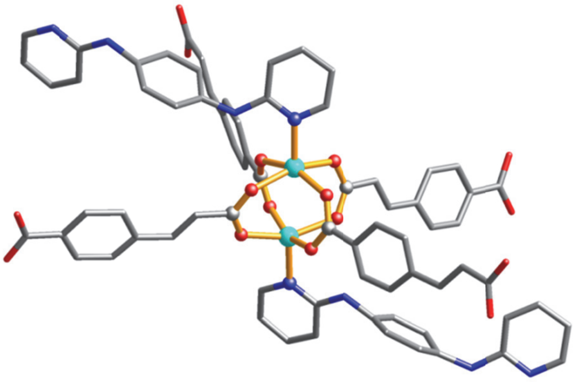

Figure S1. Coordination environment around Zn(II) center in **2**. The Zn···Zn separation in the $\{Zn_2(COO)_4\}$ SBU is 2.9694(12) Å.

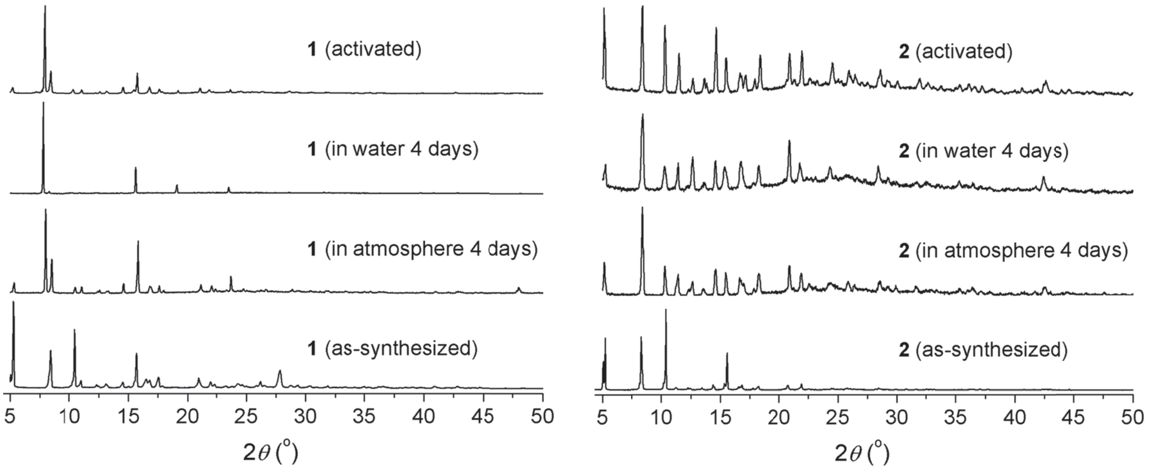

Figure S2. PXRD patterns of **1** and **2**: as-synthesized, in atmosphere 4 days, in water 4 days, and activated.

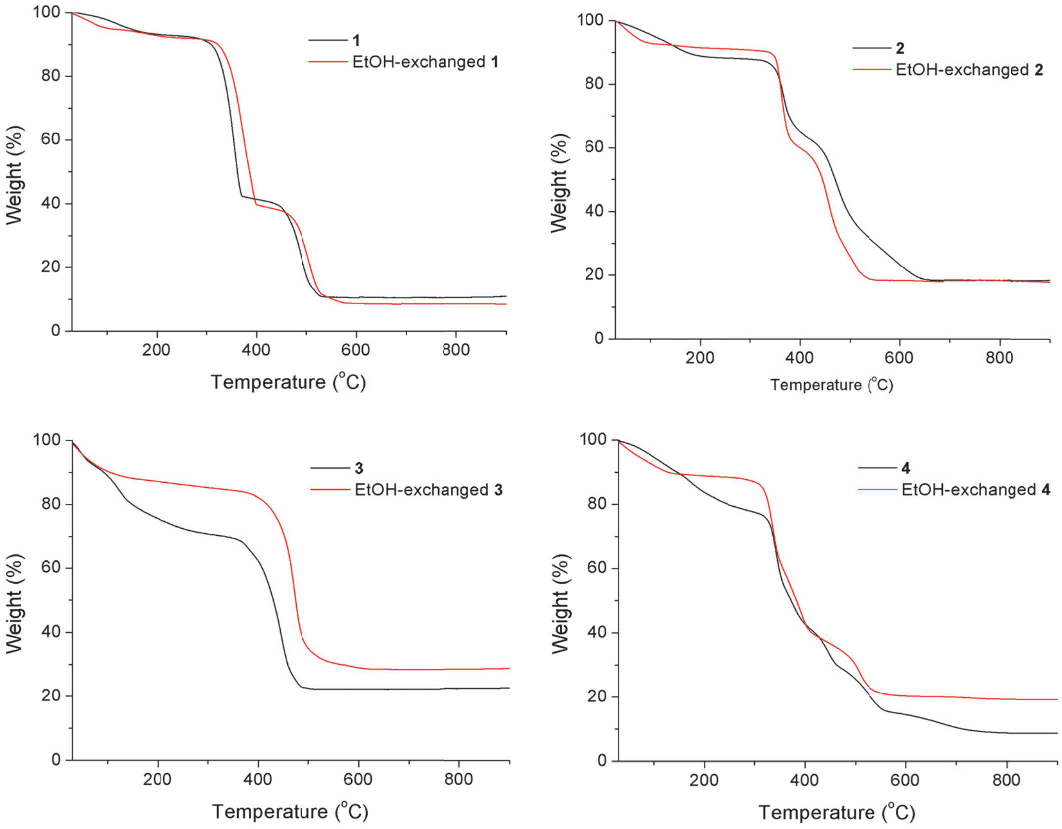

Figure S3. Thermogravimetric curves of as-synthesized and EtOH-exchanged **1**−**4**.

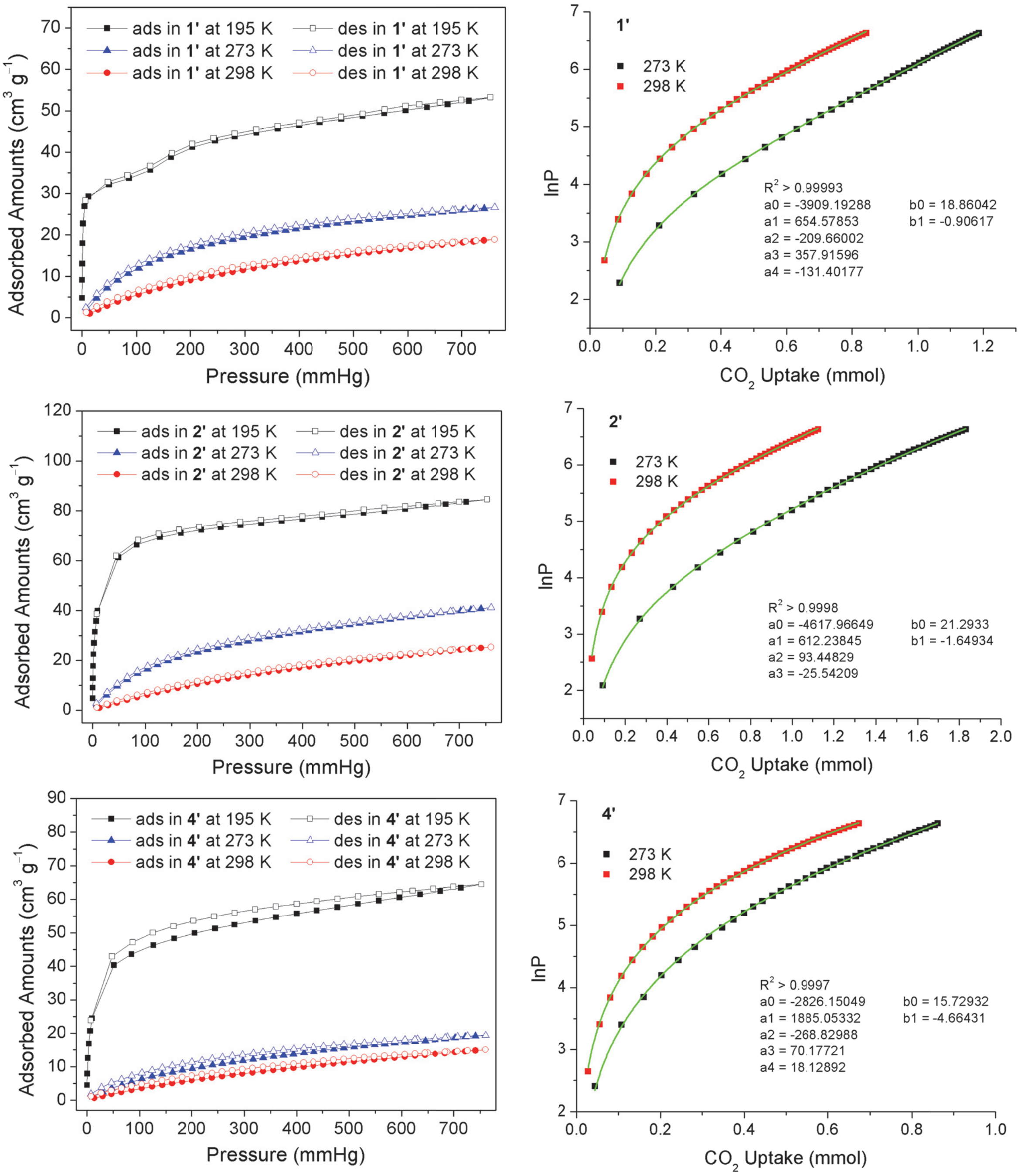

Figure S4. CO_2 adsorption isotherms and virial method fitting for Q_{st} calculation.
